# Evaluation of Camera Recognition Performance under Blockage Using Virtual Test Drive Toolchain

**DOI:** 10.3390/s23198027

**Published:** 2023-09-22

**Authors:** Sungho Son, Woongsu Lee, Hyungi Jung, Jungki Lee, Charyung Kim, Hyunwoo Lee, Hyungwon Park, Hyunmi Lee, Jeongah Jang, Sungwan Cho, Han-Cheol Ryu

**Affiliations:** 1 Department of Future Vehicle Research, Korea Automobile Testing and Research Institute, Hwaseong 18247, Republic of Korea; 1011ssh@kotsa.or.kr (S.S.);; 2 Department of Artificial Intelligence Convergence, University of Sahmyook, Seoul 01795, Republic of Korea; 3 TOD Based Transportation Research Center, University of Ajou, Suwon 16499, Republic of Korea; 4 Department of Advanced Development, Techways, Yongin 16942, Republic of Korea

**Keywords:** autonomous vehicles, camera sensor, object recognition, blockage color, object color

## Abstract

This study is the first to develop technology to evaluate the object recognition performance of camera sensors, which are increasingly important in autonomous vehicles owing to their relatively low price, and to verify the efficiency of camera recognition algorithms in obstruction situations. To this end, the concentration and color of the blockage and the type and color of the object were set as major factors, with their effects on camera recognition performance analyzed using a camera simulator based on a virtual test drive toolkit. The results show that the blockage concentration has the largest impact on object recognition, followed in order by the object type, blockage color, and object color. As for the blockage color, black exhibited better recognition performance than gray and yellow. In addition, changes in the blockage color affected the recognition of object types, resulting in different responses to each object. Through this study, we propose a blockage-based camera recognition performance evaluation method using simulation, and we establish an algorithm evaluation environment for various manufacturers through an interface with an actual camera. By suggesting the necessity and timing of future camera lens cleaning, we provide manufacturers with technical measures to improve the cleaning timing and camera safety.

## 1. Introduction

Autonomous driving is a technology that evaluates the external environment of a vehicle along with the driver’s condition and controls the vehicle based on the collected information without the direct operation of the driver. Although the commercialization of completely autonomous driving technology has yet to be implemented, Lv.3 technology has been utilized in some mass-produced vehicles. While the safety of autonomous driving has been negatively impacted because of various technical barriers, the technology has gradually developed with reinforced safety functions; however, developing Lv.4 or better technologies and ensuring driving stability requires considerable time. A partially autonomous driving technology, referred to as Lv.2, has already been commercialized and applied to mass-produced vehicles. For the commercialization of the autonomous driving phase of Lv.4 without driver intervention, consistent technology development and safety evaluations are required to improve the reliability of autonomous driving.

The three key sensors of autonomous driving are LiDAR, radar, and cameras. Various autonomous driving technologies have been developed by effectively combining the characteristics of each sensor. Object detection algorithms [1,2,3,4] have been consistently developed through the combination of cameras and LiDAR, with the limitations of these devices supplemented using the benefits of radar. The camera sensor has been essentially applied to the combination of sensors and operates as the eyes of autonomous vehicles by supplementing the limitations of LiDAR and radar sensors. A camera is a key component in automobiles, future home appliances, and robots and is the only sensor that can capture information on the texture, color, and contrast of the subject and recognize lanes and signals on roads, read signs, and classify objects, such as pedestrians, bicycles, and surrounding vehicles, with high performance. A camera sensor can obtain higher-level visual information than other sensors and is also cheaper.

When a camera detects an object in front of the vehicle, the vehicle applies automatic emergency braking and automatically keeps its lane. The camera also collects traffic information in front, maintains a distance from the vehicle ahead, recognizes traffic signs, and automatically controls high beams. Cameras have been established as major sensors for advanced driver assistance systems’ functions and have been applied in various technologies, such as forward collision prevention, lane departure avoidance, and surround view. Table 1 lists the types and quantities of the sensors used in autonomous vehicles by manufacturer.

Tesla, a representative company that realizes autonomous driving technology using only cameras, has been collecting considerable amounts of data from more than one million vehicles on the roads—a completely different and rather innovative approach, considering that autonomous driving companies, such as Waymo, that attach expensive sensors (e.g., LiDAR) to small vehicles and other companies that use cameras also apply radar; however, Mobileye, a subsidiary of Intel, is credited with first implementing autonomous vehicle camera sensor technology. Mobileye leads the development of autonomous driving technology with cameras using the EyeQ chip and has achieved significant growth to occupy more than 60% of the initiative of the camera market. Recently, however, Mobileye has been focusing on EyeQ Ultra technology that uses LiDAR and radar and has been developing technology for mass production by 2025.

Along with the technical development in the autonomous driving industry, various studies related to camera technology have been conducted for autonomous driving. First, studies on algorithms required to address environmental pollution sources caused by raindrops, snow, dust, and sand have been reported [5]. Moreover, technology for accurately measuring the pollution level of surrounding view cameras, installed on the outside and thus vulnerable to pollution, has been developed [6]. In addition, the effects of geography, environment, and weather on sensor detection have been analyzed [7], and studies on multitask learning for the adaptability of polluted sensors to recognition algorithms [8] and on filtering algorithms have been conducted [9]. A camera sensor is vulnerable to external environments, such as dust, sunlight, rain, snow, and darkness, and its performance can also be degraded by visual obstructions (blockage), such as dust, because it has smaller lens-type geometry than radar and LiDAR sensors, significantly affecting the safety of autonomous driving [10,11,12,13].

Cameras have been established as essential elements for autonomous driving; however, their pollution has severe consequences. First, in March 2018, the Tesla Model X in autonomous driving mode caused a major accident in a tunnel at a speed of approximately 60 km/h. In January 2019, an autonomous vehicle of Google Waymo hit a person, with camera pollution found to be the cause. In November 2018, an autonomous vehicle of Intel Zero Waste hit a person on the sidewalk. The cause of the accident was estimated to be the dust accumulated on the vehicle, which resulted in the pedestrian not being recognized.

Therefore, for camera sensors with performance degraded by blockage during autonomous driving, studies on the correlation between camera performance degradation and the risk of accidents should be conducted by identifying data collected from the surrounding road environment. In addition, manufacturers have attempted to propose the need for software solutions, sensor fusion, and sensor cleaning technology applications by evaluating the performance of camera recognition algorithms in solving the performance degradation by blockages.

This study developed technology to evaluate the object recognition performance of camera sensors in autonomous vehicles and verify the efficiency of the camera recognition algorithm under blockage. Through this study, the blockage effect on the camera during autonomous vehicle driving can be evaluated while identifying and supplementing the benefits and limitations of object recognition algorithms.

## 2. Research Equipment and Materials

### 2.1. Blockage-Based Camera Recognition Performance Evaluation Device

**Figure 1:**
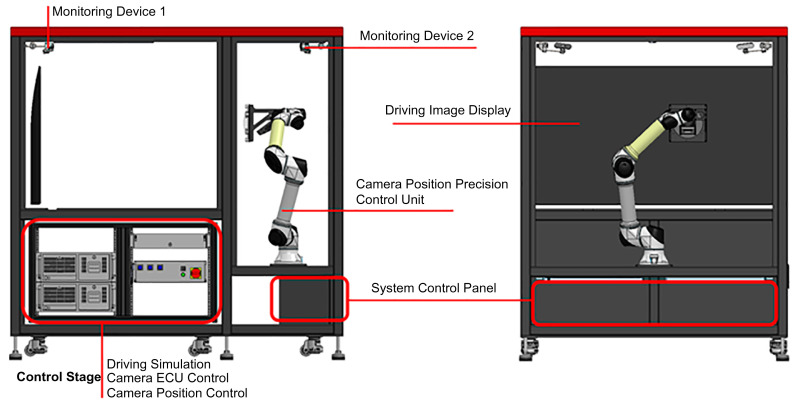
Configuration diagram of the camera evaluation device.

For this study, a blockage-based camera sensor evaluation device equipped with an actual camera that could examine the camera recognition performance using the scenario image data on the screen was developed. Figure 1 shows the configuration diagram of the device.

Table 2 shows the primary specifications of the camera simulator, comprising a darkroom test bench in which a 65-inch display with a UHD 3840 × 2160 resolution and an autonomous driving recognition camera sensor were installed. The camera simulator can perform simulations by providing static and dynamic environments to the autonomous vehicle, other surrounding vehicles, pedestrians, and other obstacles on the road. It can also perform an evaluation considering changes in the road surface conditions caused by weather and climate changes, as well as environmental factors, such as changes in the visibility range. As presented by the primary specifications in Table 2, a general-purpose test device was prepared to enable a simulation evaluation for various automotive camera sensors. In addition, a camera recognition performance evaluation environment was constructed by applying an algorithm that implements the partial occlusion of the camera view as images on the simulation image screen for virtual scenario studies on blockages.

**Figure 2:**
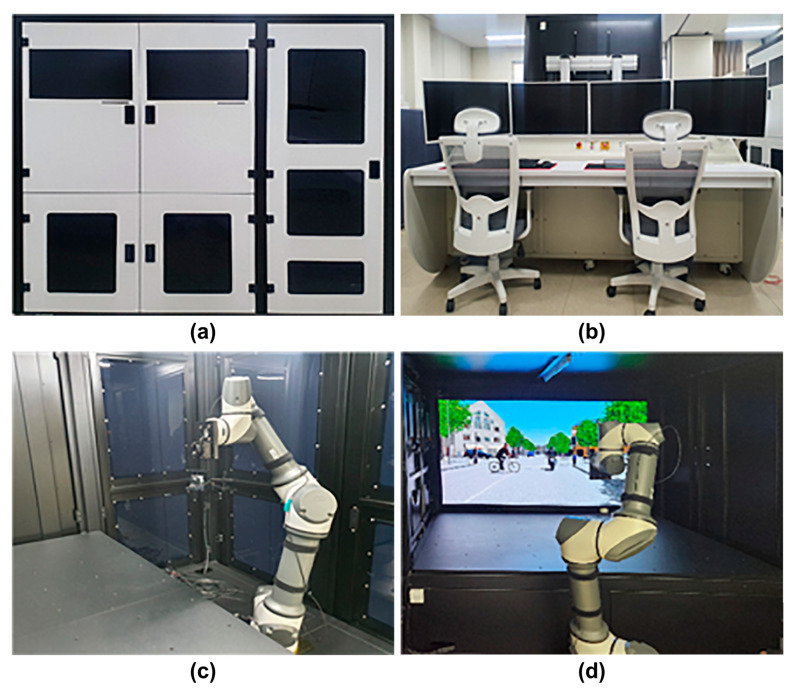
(**a**) Device exterior, (**b**) control system, (**c**) camera installation jig, and (**d**) evaluation inside screen.

The photographs of the camera evaluation device exterior, control system, and major internal components (camera installation jig and display) are shown in Figure 2.

The device performed comparison tests for blockage through virtual pollution and the pollution of the actual camera by conducting tests for the camera used in actual autonomous driving. In addition, the device facilitated the identification of camera hardware characteristics and can be expanded in the future to HiLS systems by combining it with radar and LiDAR simulators.

The V2E environment recognition sensor (camera) evaluation software was developed to evaluate whether the camera normally recognizes an object under a scenario in various blockage environments implemented by a virtual driving environment simulation. Software Blockage (ver. 1.1.1) is an algorithm that implements the partial occlusion of a screen caused by blockage on the virtual image screen recognized by the camera sensor through the software [14].

**Figure 3:**
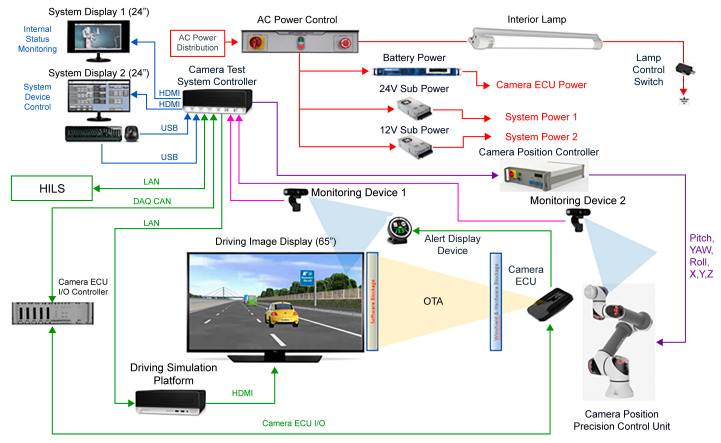
Camera evaluation system internal connection diagram.

In a test verification system, in which various cameras can be installed, the precision position control unit enables control by 0.1 mm for the normal recognition of the camera. In this cycle, the camera sensor recognizes the image and sends a signal to the camera ECU for image analysis, and control commands take approximately 100 ms. Therefore, the real-time increment was set to 100 ms or less. As the stability of recognizing images is extremely sensitive depending on the position and angle of the camera, precision position control hardware controlled with sophisticated software was implemented. The schematic of the camera evaluation system is shown in Figure 3.

### 2.2. Interface between the Camera to Be Evaluated and the Evaluation System

**Figure 4:**
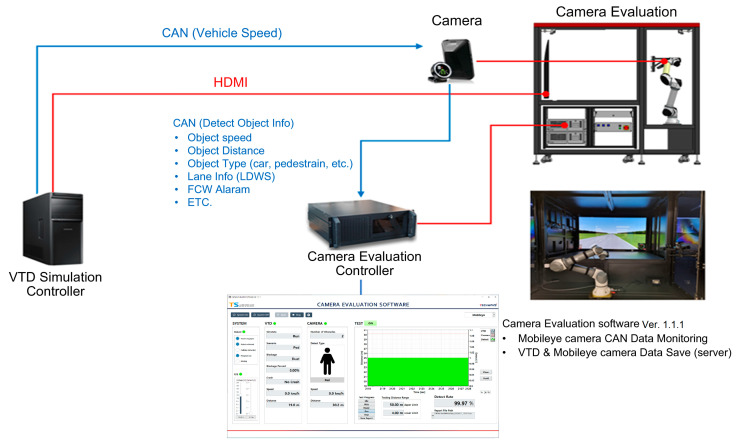
Camera interface diagram.

In a multi-camera system, the uncertainty decreases, and accuracy increases as the amount of information and the number of viewpoints increase [15]. In utilizing autonomous driving technology, the camera was selected from among companies that develop autonomous driving technology through object recognition, as well as simple signal and lane recognition [16,17]. The actual camera used for recognition in autonomous driving (Autonomous a2z, Gyeongsan-si, Gyeongsangbuk-do, Republic of Korea) was installed in the simulator. The work for the interface with the simulator shown in Figure 4 was applied as follows.

As for the coordinates of the target camera, the actual coordinates of the upper center of the indoor windshield of the actual autonomous vehicle were applied using the camera installation jig. In addition, the camera object recognition algorithm was evaluated using the camera simulator with no training on blockage or other environmental events. As for the primary specifications of the target camera, the effective pixels were 1/2.7″ 1920 × 1080, and the scan rate was 30 f/s. In addition, the AE/AWB, HDR, and LFM methods were used for image signal processing.

## 3. Research Conditions and Methods

### 3.1. Scenario Construction

**Figure 5:**
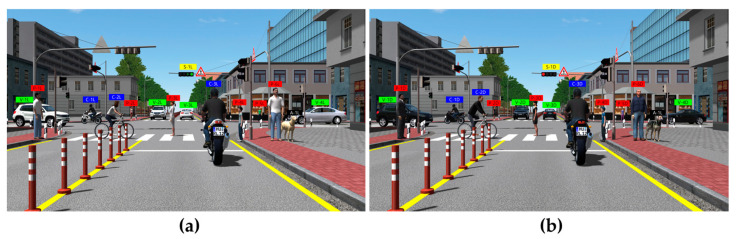
Evaluation scenario object layout: (**a**) light and (**b**) dark objects.

For scenario evaluation, road environments that are sufficiently encountered while driving were primarily selected. As shown in Table 3, pedestrians (5 adults, 1 child), vehicles (4), cyclists (3), animals (1), and signals (1) were set as objects. In addition, various arrangements were applied according to the direction and position of each object, as shown in Figure 5. The objects were classified into light and dark colors.

Here, the object score estimates the bounding box position, objectness, and class confidence of each object in deep learning-based object detection technology. Essentially, the object score is calculated as objectness × class confidence. The camera manufacturer performs object detection network learning [18,19,20,21] through various images available with open datasets and the field data acquired during autonomous driving. To analyze the trends of the data, the following mean of the object score was used.

(1) $$mean\;of\;object\;score=\frac{\sum_{1}^{60}Object\;score}{60}$$

### 3.2. Blockage Environment Implementation

**Figure 6:**
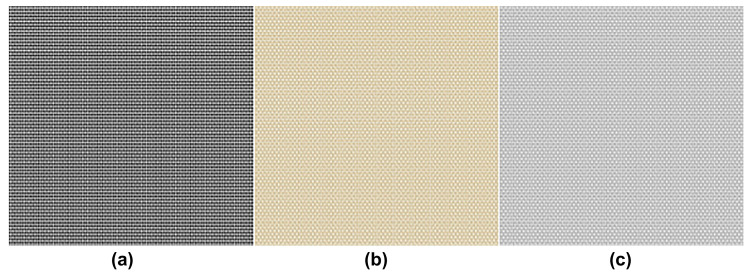
Dust color classification: (**a**) black, (**b**) yellow, and (**c**) gray.

The elements to reproduce camera blockage in a virtual environment were defined considering the factors of the weather environment, such as dust, snow, and rain. In this study, focus was given to dust. Based on the actual road environment of Korea, (a) black, which expresses dust mixed with emissions, asphalt, and tire dust on roads (Figure 6a), (b) yellow, which expresses yellow dust (Figure 6b), and (c) gray, which expresses cement and stone powder that constitute roads and structures (Figure 6c) were selected as representative dust color classifications.

To create the dust images, a particle size of 75 μm was applied within the actual Arizona dust size range. When the dust was in contact with the camera lens, dust images were created considering the distance between the lens and monitor and the resolution of the actual monitor.

**Figure 7:**
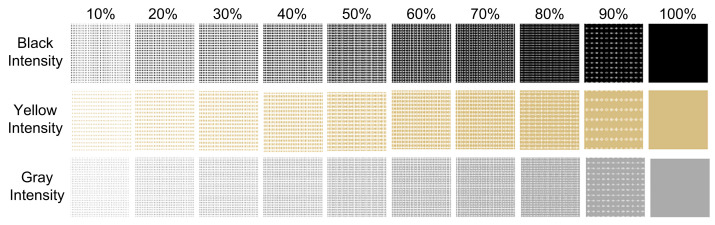
Change by dust concentration.

As shown in Figure 7, a dust concentration of up to 70% was applied at 5% intervals for the three colors. The dust concentration was determined by calculating the empty area and dust area by pixel, with the entire angle of view of the camera constructed with the same pattern.

**Figure 8:**
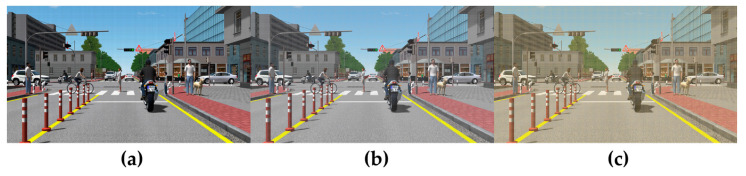
Dust coverage scenarios: (**a**) black 5%, (**b**) gray 25%, and (**c**) yellow 40%.

The created dust was loaded onto the scenario screen, and evaluation scenarios were constructed by changing the blockage concentration and color, as shown in Figure 8 [22].

### 3.3. Data Characteristics

For data collection, the constructed scenarios were observed on the screen through the camera angle. The camera object recognition algorithm was evaluated by changing the blockage conditions by increasing the concentration of the three colors by 5%. The data were collected by applying the real-time camera object recognition algorithm under static scenarios. Two normal data colors were added to two object colors, the three dust colors, and fourteen dust concentrations from 5 to 70%. A total of 1290 items were used for 15 individual objects. As 60 data fragments were collected for each item, a total of 77,400 data were collected and analyzed for each object. The object score that expressed the confidence value of recognizing the object as a score for each item was set as the dependent variable to analyze the results.

To extract the individual characteristics of each variable, data collection was performed in a similar situation by reflecting real conditions rather than controlled experimental conditions. Among the four factors of the data to be analyzed, the object factors (15) and concentration variables (14) exhibited many levels. Nonlinear relationships occurred for three factors except for the concentration variable. Limitations in generalizing and expressing statistical models on complex interactions between the variables occurred. To represent the difference in object score depending on the variables, the statistical methods were mostly designed for inference and estimation, with some hypotheses and restrictions necessary before model application, which explains the phenomenon by interpreting the relationship between the estimator and explanatory variable because it performs prediction on a strictly mathematical basis but cannot reflect complex interactions between many variables that affect the dependent variable.

Unlike statistical methods, machine learning aims at prediction rather than interpretation and can handle complex and highly nonlinear relationships between dependent and independent variables. As it is faster and simpler than statistical models, important factors were rapidly extracted by examining the importance of variables using random forest. In addition, the analysis of variance (ANOVA) method was used to verify the statistical significance of the difference in the object score depending on the three groups of the blockage color and two groups of the object color.

## 4. Results and Discussions

### 4.1. Analysis of the Importance of the Major Variables

Machine learning can handle complex and highly nonlinear relationships between dependent and independent variables. As this method randomly selects both data and variables in each tree, each tree has diverse and abundant expressions as an uncorrelated model (≒independent). For random forest parameter optimization, hyperparameters were entered in sequence, GridSearchCV was used to derive the optimal parameters [23], and the optimal model was constructed using these hyperparameters.

Ensemble models, such as Random Forest, which belongs to the tree-based family of models, generally provide a measure called feature importance, which can be interpreted as the importance of the variables or features. The underlying mechanism is based on the concept of reducing impurity through information gain. In decision trees, nodes are split using a method that minimizes impurity. In ensemble methods composed of multiple decision trees, variable importance is determined by averaging the importance values from each tree [24].

(2) $$I_{jm}=\sum_{R:j\in R\in t_{m}}\left[\frac{nR}{n}Imp\left(R\right)-\frac{nR_{left}}{n}Imp\left(R_{left}\right)-\frac{nR_{right}}{n}Imp\left(R_{right}\right)\right]$$

Here, nR represents the number of samples at node *R*, and $\left\{R:j\in R\in t_{m}\right\}$ denotes the set of nodes in the individual tree $t_{m}$ where the *j*-th variable is chosen for splitting. Additionally, $R_{left}$ and $R_{right}$ refer to the left and right nodes of node *R*, respectively.

The importance $I_{j}$ for the *j*-th variable is calculated as follows.

(3) $$I_{j}=\frac{1}{M}\sum_{m=1}^{M}I_{jm}$$

Finally, it is transformed through normalization as follows.

(4) $${I^{\prime }}_{j}=\frac{I_{j}}{\sum_{k}I_{k}}$$

**Figure 9:**
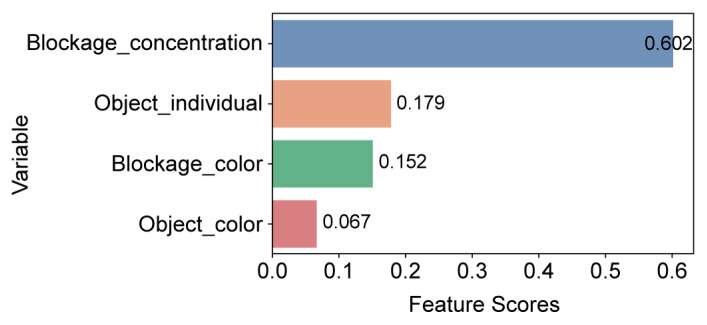
Results of feature importance.

The feature importance analysis results show that the blockage concentration (feature importance value: 0.602) has the largest impact on object recognition, followed by the object type (0.179), blockage color (0.152), and object color (0.067), as shown in Figure 9. For the concentration variable with the highest feature importance, a certain concentration range was specified to examine its impact on the remaining variables.

### 4.2. Analysis of the Effects of the Object and Dust Colors on Object Recognition

The blockage concentration, which has the largest impact on object recognition, should be specified to analyze the effects of dust and object colors on object recognition. Considering the characteristics of blockage that gradually accumulated from the clean condition, the effects of the blockage and object colors on the recognition rate were examined based on a 5% dust concentration, which is the lowest concentration in this study and the most easily accessible in the initial stage of pollution.

**Figure 10:**
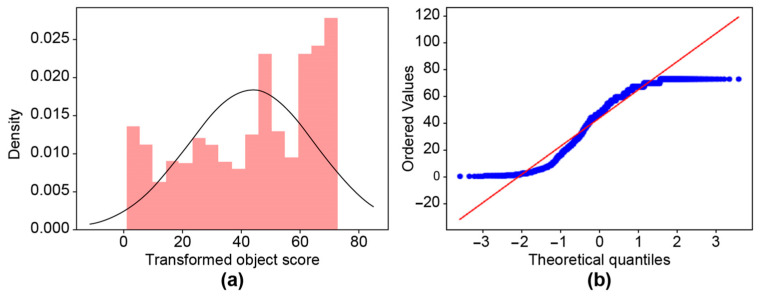
Box–Cox transformation for blockage at 5%: (**a**) histogram and (**b**) QQ plot.

A histogram and QQ plot were drawn to verify the normality of the data, but bias was detected. To convert the distribution into a normal distribution, “Box–Cox”, a function conversion method that adjusts the skew of the distribution, was used [25]. Consequently, the data distribution was normalized by adjusting the lambda value in Equation (5) and searching for the optimal lambda value at which the distribution became a normal distribution, as shown in Figure 10.

(5) $${y_{i}}^{\lambda }=\left\{\begin{array}{c} \frac{{y_{i}}^{\lambda }-1}{\lambda }\;if\;\lambda \neq 0,\\ \ln{(y}_{i})\;if\;\lambda =0 \end{array}\right.$$

Using six factors from three dust and two object colors, descriptive statistics that could identify differences between the individual groups were applied for 4172 data in the 5% blockage concentration range. Table 4 shows the average and standard deviation of each group.

**Figure 11:**
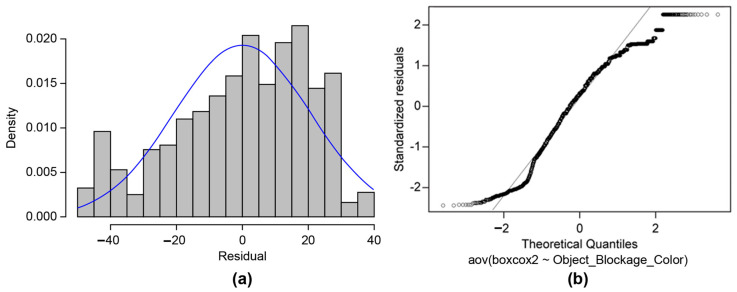
Residual analysis for normality test for six groups: (**a**) histogram and (**b**) QQ plot.

The normality was satisfied in the Shapiro–Wilk normality test results (W = 0.958, *p* < 2.2 × 10^−16^), and the histogram and Q-Q plot for the residual analysis for the six groups, which show the difference between the Box–Cox conversion values and representative values, are shown in Figure 11. As the skewness value was −0.4276 and the kurtosis value was 1.9385, normality was assumed [26], and an ANOVA analysis was conducted.

Levene’s test of variance homogeneity showed significant results (Levene statistic 38.366, *p* < 0.05), indicating that the variances of the six groups were different. As homoscedasticity was not satisfied, Welch and Brown–Forsythe mean tests were conducted on the groups. Table 5 shows the robust test results for the homogeneity of means. The significance probability confirmed significant differences among the six groups [27].

Table 6 shows Dunnett’s T3 post hoc test results for fifteen items among the six groups.

**Figure 12:**
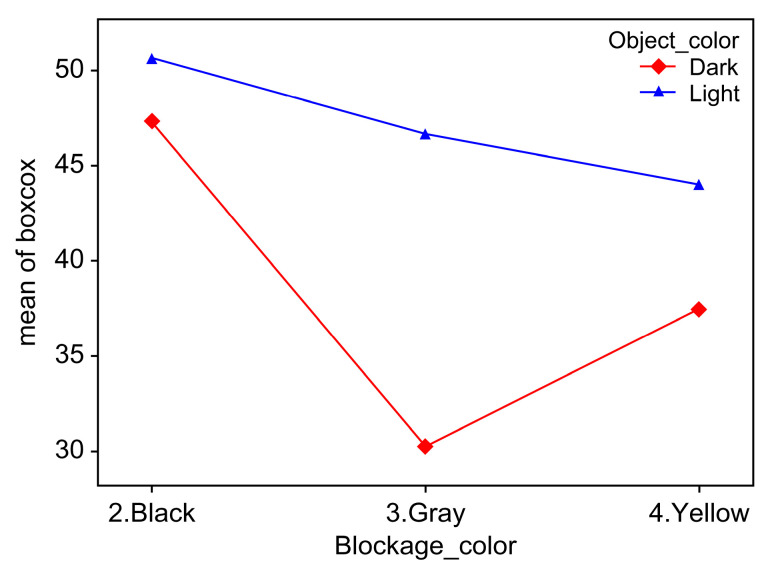
Perception score changes according to dust and object colors.

Black_Dark was −3.33 lower than Black_Light but was higher than Gray_Dark (17), Yellow_Dark (9.89), and Yellow_Light (3.32); therefore, Black_Light exhibited the highest object score, followed by Black_Dark, Gray_Light, Yellow_Light, Yellow_Dark, and Gray_Dark. In the post hoc test results, most of the mutual significance results of the six factors were satisfied. The significance probability between Black_Dark and Gray_Light and that between Gray_Light and Yellow_Light exceeded 0.05, implying that the average difference between the two groups was not significant, as shown in Figure 12.

As shown in Figure 12, the effects of the dust and object colors on the recognition rate can be visually confirmed. Overall, the light objects exhibited higher object scores than the dark objects. This shows that the camera recognition algorithm is insufficient in recognition learning for dark objects compared to bright objects. For the colors, high Box–Cox conversion mean scores were observed for black blockage regardless of the object color. Basically, camera object recognition learning involves object recognition in the nighttime state; therefore, it seems to be a result of the darkness of the nighttime state being similar to the effect of black dust. In addition, the fact that black dust had a particularly high recognition score for certain objects, such as signals and cyclists, also had an impact. Because gray and yellow are relatively bright, contrasting dark objects showed lower recognition scores than bright objects. In particular, the combination of blockage gray and a dark object color exhibited the lowest object score. For the camera object recognition algorithm, improvements are needed to increase the recognition rate for dark objects and gray and yellow dust.

### 4.3. Effects of Blockage on Different Objects

Under static scenarios, five object types were distinguished, with various object shapes arranged at different positions for each object type. The blockage concentration was increased by 5% to 70%.

**Figure 13:**
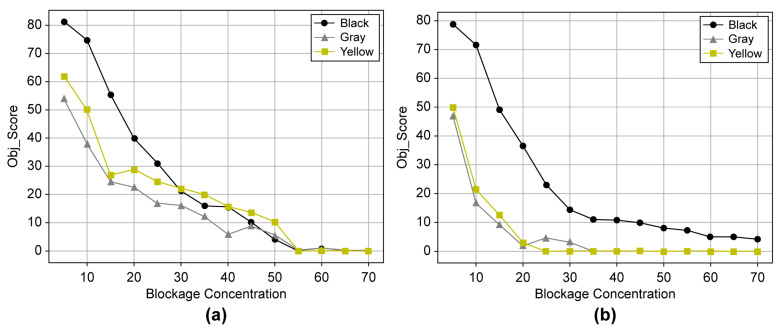
(**a**) Light object and (**b**) dark object mean scores by concentration.

**Figure 14:**
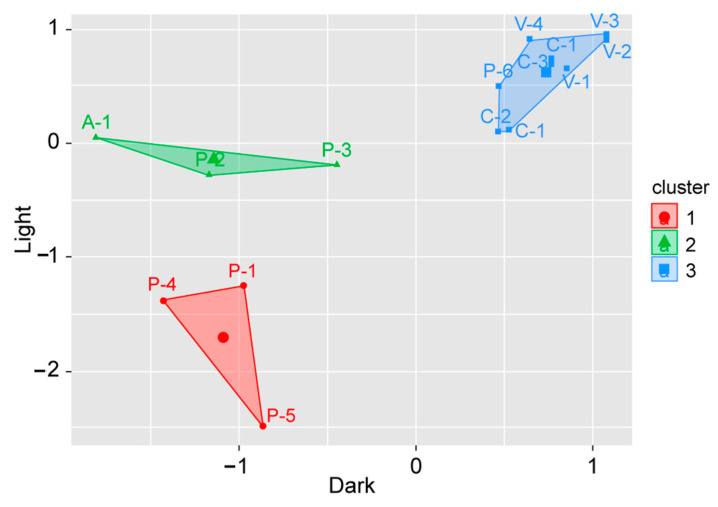
K-means clustering results.

Figure 13 shows the object score by object color according to the blockage concentration for all the data. Notably, a difference in the object score was observed depending on the dust color for a concentration up to 25%. An analysis was conducted for the 5–25% concentration range to examine the object score tendency by object type according to the blockage concentration. As a large difference in the object score of each object in the normal state was observed, K-means clustering was conducted on the object scores of light and dark object colors for 15 objects to classify objects with similar patterns into groups [28,29]. The initial number of clusters was set to three. Figure 14 shows the final clustering result.

For the objects in the green group (A-1, P-2, and P-3), the score was low when the object color was dark and relatively high when it was light. For the objects in the red group (P-1, P-4, and P-5), the object score was low regardless of the light and dark object colors. In the case of the objects in the blue group (C-1, C-2, C-3, P-6, V-1, V-2, V-3, V-4, and S-1), the object score was high regardless of the light and dark object colors.

For pedestrians, the object size affected the object score. For P-1, the overall object score was low under the influence of the background (V-1 and traffic signal). The vehicles, cyclists, and signals mostly exhibited high object scores regardless of color.

**Figure 15:**
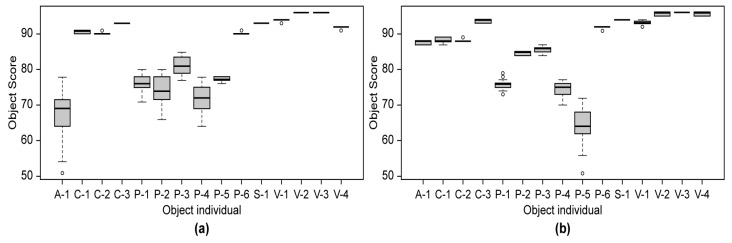
(**a**) Normal state dark object score distribution and (**b**) normal state light object score distribution table.

To examine the effect of blockage on the recognition of object types, the object score values in the normal state without blockage were summarized for 30 objects, as shown in Figure 15.

**Figure 16:**
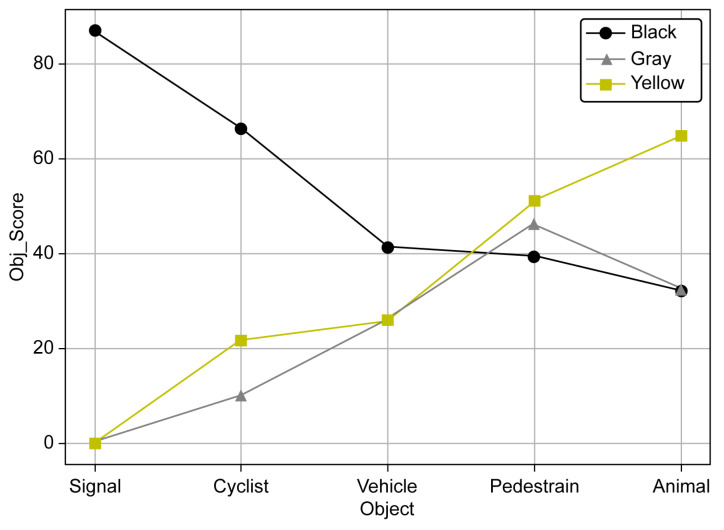
Recognition score by object and blockage color at 5–25% blockage.

The effects of the blockage concentration on the object types were analyzed for 12 objects (dark: C-1, C-2, P-6, S-1, and V-4; light: A-1, C-1, C-2, P-3, P-6, S-1, and V-1), corresponding to object scores of 85–93 with little difference in the individual object scores, as shown in Figure 15. The twelve objects were grouped into five object types. Figure 16 shows the object score trend in the 5–25% concentration range.

**Figure 17:**
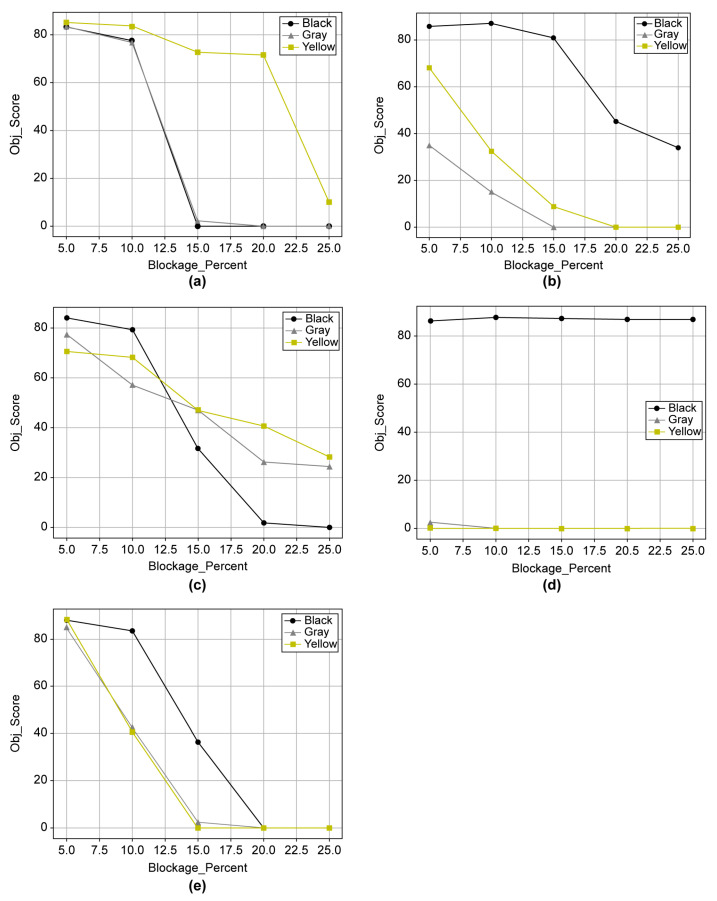
Recognition score by object at 5–25% blockage: (**a**) animal, (**b**) cyclist, (**c**) pedestrian, (**d**) signal, and (**e**) vehicle.

A noticeable difference in the object score between black and both gray and yellow was observed. For black, the object score decreased in the order of the signal, cyclist, vehicle, pedestrian, and animal. For yellow, it decreased in the order of the animal, pedestrian, vehicle, cyclist, and signal. For gray, it decreased in the order of the pedestrian, animal, vehicle, cyclist, and signal. Compared with black, yellow and gray exhibited almost the opposite object score results. In other words, object recognition was significantly affected by the blockage color. Particularly, for the signal, the pollution resistance was high for black but was significantly low for gray and yellow. The signal exhibited the largest difference in recognition tendency depending on the color, followed by the cyclist and vehicle. Pedestrians and vehicles, which can be considered representative objects, had object scores within similar categories regardless of color. Since learning from various cases was greater than that of other objects, it does not seem to have been greatly affected by blockage color. Nevertheless, the yellow and gray colors need improvement, particularly for the low object scores of the signal and cyclist. Figure 17 shows the object scores of five individual objects with respect to the blockage concentration.

For the animal, black and gray exhibited similar tendencies, with object recognition failing at a blockage concentration of 15%. For yellow, recognition was impossible at 25%. For the cyclist, the object score sharply decreased from 20% for black but was maintained at approximately 40. For yellow and gray, object recognition failed between 15–20%. For the pedestrian, the object score slowly decreased from approximately 70 to 20 as the blockage concentration increased for gray and yellow. For black, it sharply decreased from 10% to 15%, and object recognition was infeasible at 20%. For the signal, a noticeable difference in the object score between black and both gray and yellow was observed. Particularly, for gray and yellow, the signal recognition was almost infeasible from 5%, indicating that the algorithm for blockage needs to be supplemented for this range in the future. Finally, in the case of the vehicle, no significant difference in color compared with other objects was observed, with recognition failing at 15% to 20%. Based on these results, when the manufacturer managed the object score at 40 or higher in the case of 10% blockage, 11 objects out of 15 met the management target; however, for the remaining four objects, the recognition algorithm needed to be improved through learning. In terms of autonomous driving sensor cleaning, this 10% blockage can be presented as the basis for camera lens cleaning to remove blockages.

## 5. Conclusions

In this study, the object recognition performance of an autonomous driving camera algorithm was evaluated from different angles by applying virtual blockages. The implications and limitations derived from the research results are as follows.

First, the concentration of the blockage was the most significant factor affecting object recognition. When the blockage concentration was 10% or less, most objects could be accurately recognized. If there is no additional improvement in cognitive performance owing to dust contamination, the standard for cleaning the camera is when contamination exceeds 10% concentration. Therefore, the appropriate timing of camera lens cleaning could be determined.

Second, as for the blockage color, yellow and gray were found to be more unfavorable for object recognition than black, indicating different tendencies depending on the object type. For important objects, such as traffic signals, the recognition algorithm needed to be improved.

Third, through the developed interface between the evaluation system and autonomous driving camera, a general-purpose methodology capable of comparing and evaluating the performance of camera algorithms from various manufacturers in virtual environments was presented.

Fourth, the costs and risks could be reduced by reproducing vehicle driving situation cases not easily implementable in a virtual environment. In addition, various scenarios other than dust, such as weather conditions, pedestrians, vehicles, and buildings, could be utilized for camera algorithm training.

Fifth, autonomous driving camera recognition technology manufacturers should set and comply with reference points for object recognition even at a certain level of blockage during technical development and mass production; these reference points could improve the safety of autonomous driving technology and user confidence.

In this study, we investigated object recognition based on dust concentration and color. In the future, performance evaluations of various cameras are necessary. The type of camera covering must additionally consider the impact of not only dust, but also weather conditions, such as fog and rain. In addition, studies on the recognition rate depending on the background and object colors and differences by object type will be conducted by arranging road environments and objects in a strictly controlled environment. In addition, an experiment in which dust is sprayed onto an actual camera and compared with the dust in a virtual environment will be conducted to enhance the validity of the virtual environment test. Finally, studies on the comparison of the performances of various camera manufacturers and an analysis of the differences depending on blockage will be conducted.

## Figures and Tables

**Table 1:** Perception sensor usage status by major manufacturers.

Maker	Camera	Radar	LiDAR	Total
Tesla (Austin, TX, USA)	8	0	0	8
AutoX (Shenzhen, China)	8	6	1	15
Pony.ai (Fremont, CA, USA)	7	4	4	15
Baidu (Beijing, China)	6	4	5	15
Waymo (San Francisco, CA, USA)	8	6	4	18
Mobileye (New York, NY, USA)	11	6	3	20
Aptiv (Hanover, Ireland)	2	10	9	21
Sony (Tokyo, Japan)	16	5	4	25
NVIDIA (Santa Clara, CA, USA)	14	9	3	26
Cruise (San Francisco, CA, USA)	14	21	5	40

**Table 2:** Primary specifications of the camera evaluation device.

Manufacturer	Techways (Yongin-si, Gyeonggi-do, Republic of Korea)
Dimension (W) × (D) × (H)	1800 mm × 1600 mm × 2000 mm
Driving display	65″ UHD (3840 × 2160), 120 f/s
System display	24″ internal status monitoring, 24″ device control
Camera	Autonomous a2z
Camera position precision control unit	Pitch, yaw, roll, X, Y, Z
Camera controller	Camera ECU, I/O controller
Power	AC power control, DC 12V, 24V sub-power

**Table 3:** Classification of objects.

Object Type		Symbol	No. Objects
Pedestrian	Adult	P-1	6
		P-2	
		P-3	
	Child	P-4	
	Adult	P-5	
		P-6	
Vehicle		V-1	4
		V-2	
		V-3	
		V-4	
Cyclist	Motorcycle	C-1	3
	Bicycle	C-2	
	Motorcycle	C-3	
Animal		A-1	1
Traffic signal		S-1	1
Total no. objects			15

**Table 4:** Descriptive statistics table for dust and object colors.

Blockage Color_Object Color	N	Average	Standard Deviation	Standard Error	95% Confidence Interval		Min	Max
					Low	High		
Black_Dark	855	47.3610	19.88092	0.67991	46.0265	48.6954	1.65	72.83
Black_Light	866	50.6915	19.43979	0.66059	49.3950	51.9881	0.93	72.83
Gray_Dark	580	30.2051	18.53614	0.76967	28.6934	31.7168	0.93	70.02
Gray_Light	593	46.6915	22.88584	0.93981	44.8457	48.5372	0.93	72.83
Yellow_Dark	574	37.4648	18.88618	0.78829	35.9165	39.0131	1.33	70.02
Yellow_Light	704	44.0406	24.03756	0.90595	42.2619	45.8193	1.05	72.83
Total	4172	43.6503	21.73887	0.33656	42.9904	44.3101	0.93	72.83

**Table 5:** Robust test for homogeneity of means.

Method	Statistics	Degree of Freedom (between Groups)	Degree of Freedom (within Groups)	Classical Test Theory Significance Probability
Welch	103.595	5	1858.401	<0.001
Brown–Forsythe	87.159	5	3825.313	<0.001

**Table 6:** Games–Howell multiple comparison table for dust and object colors. (* : Significant data notation).

(I) Blockage Color_Object Color	(J) Blockage Color_Object Color	Average Difference (I − J)	Standard Error	Classical Test Theory Probability	95% Confidence Interval	
					Low	High
Black_Dark	Black_Light	−3.33056 *	0.94798	0.006	−6.0351	−0.6260
Black_Dark	Gray_Dark	17.15586 *	1.02697	<0.001	14.2249	20.0868
Black_Dark	Gray_Light	0.66948	1.15997	0.993	−2.6416	3.9806
Black_Dark	Yellow_Dark	9.89612 *	1.04100	<0.001	6.9250	12.8672
Black_Dark	Yellow_Light	3.32037 *	1.13271	0.040	0.0879	6.5529
Black_Light	Gray_Dark	20.48642 *	1.01428	<0.001	17.5916	23.3812
Black_Light	Gray_Light	4.00004 *	1.14875	0.007	0.7208	7.2793
Black_Light	Yellow_Dark	13.22668 *	1.02849	<0.001	10.2912	16.1621
Black_Light	Yellow_Light	6.65092 *	1.12122	<0.001	3.4512	9.8507
Gray_Dark	Gray_Light	−16.48638 *	1.21476	<0.001	−19.9540	−13.0187
Gray_Dark	Yellow_Dark	−7.25974 *	1.10173	<0.001	−10.4046	−4.1148
Gray_Dark	Yellow_Light	−13.83549 *	1.18876	<0.001	−17.2283	−10.4427
Gray_Light	Yellow_Dark	9.22664 *	1.22664	<0.001	5.7251	12.7282
Gray_Light	Yellow_Light	2.65088	1.30537	0.325	−1.0747	6.3765
Yellow_Dark	Yellow_Light	−6.57575 *	1.20090	<0.001	−10.0032	−3.1483

## Data Availability

Not applicable.

## References

[1] An P., Liang J., Yu K., Fang B., Ma J. (2022). Deep Structural Information Fusion for 3D Object Detection on LiDAR—Camera System. Comput. Vis. Image Underst..

[2] Chen M., Liu P., Zhao H. (2023). LiDAR-Camera Fusion: Dual Transformer Enhancement for 3D Object Detection. Eng. Appl. Artif. Intell..

[3] Liu L., He J., Ren K., Xiao Z., Hou Y. (2022). A LiDAR—Camera Fusion 3D Object Detection Algorithm. Information.

[4] Yeong J., Velasco-Hernandez G., Barry J., Walsh J. (2021). Sensor and Sensor Fusion Technology in Autonomous Vehicles: A Review. Sensors.

[5] Das A. (2019). SoildNet: Soiling Degradation Detection in Autonomous Driving. arXiv.

[6] Uřičář M., Křížek P., Sistu G., Yogamani S. SoilingNet: Soiling Detection on Automotive Surround-View Cameras. Proceedings of the IEEE Intelligent Transportation Systems Conference (ITSC).

[7] Kenk M.A., Hassaballah M. (2008). DAWN: Vehicle Detection in Adverse Weather Nature Dataset. arXiv.

[8] Yu F., Chen H., Wang X., Xian W., Chen Y., Liu F., Madhavan V., Darrell T. BDD100K: A Diverse Driving Dataset for Heterogeneous Multitask Learning. Proceedings of the IEEE/CVF Conference on Computer Vision and Pattern Recognition (CVPR).

[9] Agunbiade Y.O., Dehinbo J.O., Zuva T., Akanbi A.K. (2018). Road Detection Technique Using Filters with Application to Autonomous Driving System. arXiv.

[10] Freimuth H., König M. (2019). A Framework for Automated Acquisition and Processing of as-Built Data with Autonomous Unmanned Aerial Vehicles. Sensors.

[11] Mohd Ansari Shajahan J., Mamani Reyes S., Xiao J. Camera Lens Dust Detection and Dust Removal for Mobile Robots in Dusty Fields. Proceedings of the IEEE International Conference on Robotics and Biomimetics (ROBIO).

[12] Huang Z.-Y., Lai Y.-C. Image-Based Sense and Avoid of Small Scale UAV Using Deep Learning Approach. Proceedings of the International Conference on Unmanned Aircraft Systems (ICUAS).

[13] Premebida C., Monteiro G., Nunes U., Peixoto P. A Lidar and Vision-Based Approach for Pedestrian and Vehicle Detection and Tracking. Proceedings of the IEEE Intelligent Transportation Systems Conference.

[14] Wu X., Wang L. Camera Simulator for Benchmarking Computational Photography Algorithms. Proceedings of the IEEE Conference on Computer Vision and Pattern Recognition Workshops.

[15] Kim K., Davis L.S. Multi-camera Tracking and Segmentation of Occluded People on Ground Plane Using Search-Guided Particle Filtering. Proceedings of the Computer Vision—ECCV 2006: 9th European Conference on Computer Vision.

[16] Arulkumar V., Aruna M., Lakshmi M.A., Rao B.H. Super Resolution and Demosaicing Based Self Learning Adaptive Dictionary Image Denoising Framework. Proceedings of the 2021 5th International Conference on Intelligent Computing and Control Systems (ICICCS).

[17] He K., Zhang X., Ren S., Sun J. Deep Residual Learning for Image Recognition. Proceedings of the IEEE Conference on Computer Vision and Pattern Recognition.

[18] Du K., Bobkov A. (2023). An Overview of Object Detection and Tracking Algorithms. Eng. Proc..

[19] Ma L., Meng D., Zhao S., An B. (2023). Visual Localization with a Monocular Camera for Unmanned Aerial Vehicle Based on Landmark Detection and Tracking Using YOLOv5 and DeepSORT. Int. J. Adv. Robot. Syst..

[20] Ghaderzadeh M., Aria M., Hosseini A., Asadi F., Bashash D., Abolghasemi H. (2022). A Fast and Efficient CNN Model for B-ALL Diagnosis and its Subtypes Classification Using Peripheral Blood Smear Images. Int. J. Intell. Syst..

[21] Garavand A., Behmanesh A., Aslani N., Sadeghsalehi H., Ghaderzadeh M. (2023). Towards Diagnostic Aided Systems in Coronary Artery Disease Detection: A Comprehensive Multiview Survey of the State of the Art. Int. J. Intell. Syst..

[22] Hosseini A., Eshraghi M.A., Taami T., Sadeghsalehi H., Hoseinzadeh Z., Ghaderzadeh M., Rafiee M. (2023). A Mobile Application Based on Efficient Lightweight CNN Model for Classification of B-ALL Cancer from Non-Cancerous Cells: A Design and Implementation Study. Inform. Med. Unlocked.

[23] Pedregosa F., Varoquaux G., Gramfort A., Michel V., Thirion B., Grisel O., Blondel M., Prettenhofer P., Weiss R., Dubourg V. (2011). Scikit-Learn: Machine Learning in Python. J. Mach. Learn. Res..

[24] Rogers J., Gunn S., Saunders C., Grobelnik M., Gunn S., Shawe-Taylor J. (2005). Identifying Feature Relevance Using a Random Forest. Subspace, Latent Structure and Feature Selection.

[25] Guo Y., Yang Y. (2002). Improved Box-Cox Transformation for Non-normal Data. Stat. Probab. Lett..

[26] Hong S., Malik M.L., Lee M.-K. (2003). Testing Configural, Metric, Scalar, and Latent Mean Invariance Across Genders in Sociotropy and Autonomy Using a Non-western Sample. Educ. Psychol. Meas..

[27] Cheddad A. (2020). On Box-Cox Transformation for Image Normality and Pattern Classification. IEEE Access.

[28] Hautamäki V., Pöllänen A., Kinnunen T., Lee K.A., Li H., Fränti P. (2014). A Comparison of Categorical Attribute Data Clustering Methods. Structural, Syntactic, and Statistical Pattern Recognition: Joint IAPR International Workshop, S+.

[29] von Luxburg U. (2007). A Tutorial on Spectral Clustering. Stat. Comput..

